# Ursolic acid-mediated apoptosis of K562 cells involves Stat5/Akt pathway inhibition through the induction of Gfi-1

**DOI:** 10.1038/srep33358

**Published:** 2016-09-16

**Authors:** Ze Lin, Jikai Jiang, Xiao-Shan Liu

**Affiliations:** 1 Department of Biochemistry, Shantou University Medical College, No. 22 Xinlin Road, Jinping District, Shantou, 510451, China; 2 Department of Biochemistry, Guangzhou Medical University, Xinzao Town, Panyu District, Guangzhou 511436, China

## Abstract

Ursolic acid (UA) is a promising natural compound for cancer prevention and therapy. We previously reported that UA induced apoptosis in CML-derived K562 cells. Here we show that the apoptotic process is accompanied by down-regulation of Bcl-xL and Mcl-1 expression and dephosphorylation of Bad. These events are associated with Stat5 inhibition, which is partially mediated through elevated expression of transcriptional repressor Gfi-1. Gfi-1 knockdown using siRNA abrogates the ability of UA to decrease Stat5b expression and attenuates apoptosis induction by UA. We also demonstrate that UA suppresses the Akt kinase activity by inhibiting Akt1/2 expression, which correlates with Stat5 inhibition. Stat5 activity inhibited by a chemical inhibitor or siRNA, Akt1/2 mRNA expression is suppressed. Moreover, we show that UA exerts growth-inhibition in Imatinib-resistant K562/G01. UA has synergistic effects when used in combination with Imatinib in both K562 and K562/G01. Altogether, the data provide evidence that UA’s pro-apoptotic effect in K562 cells is associated with the Gfi-1/Stat5/Akt pathway. The findings indicate that UA could potentially be a useful agent in the treatment of CML.

Chronic myelogenous leukemia (CML) is a myeloproliferative disorder. Most cases of CML are characterized by the presence of the Philadelphia chromosome, the reciprocal translocation of chromosomes 9 and 22 that generates the bcr–abl fusion gene encoding a constitutively active p210 Bcr–Abl tyrosine kinase^1^,^2^. Currently, Bcr/Abl kinase inhibition by Imatinib or more potent Dasatinib and Nilotinib is considered standard therapy for CML. However, resistance or intolerance to these tyrosine kinase inhibitors (TKI) is often encountered^3^,^4^. Therefore, seeking alterative targets and additional therapeutic strategies for CML is of intense interest.

Stat5, including two highly related proteins Stat5a and Stat5b, belong to a family of cytoplasmic transcription factors. Stat5 is widely expressed in normal tissues and can be activated by a variety of cytokines and growth factors^5^. Over the past decade, the involvement of the Stat5 signaling in leukemogenesis in Bcr/Abl positive CML cells has been extensively investigated^6^,^7^,^8^. Stat5 is highly expressed and constitutively activated in CML cells^9^. Elevated Stat 5 mRNA has also been demonstrated to counteract TKI therapy in CML^10^,^11^. Stat5 knockdown by dominant-negative mutants or siRNA can reduce the survival of p210 Bcr–Abl positive cells and induce apoptosis^12^,^13^,^14^. These suggest that a drug targeting Stat5 may have important implications for CML therapy^15^. Much interest has recently been generated in the development of compounds that inhibit Stat5 activity. For example, pimozide was identified as a Stat5 inhibitor with significant activity against CML^16^. TR120 was discovered to induce apoptosis in CML cell lines by reducing Stat5 expression^17^.

Plant-derived compounds are increasingly considered as a major source of anticancer drugs. Ursolic acid (UA) is a pentacyclic triterpene acid naturally occurring in food and medicinal herbs. UA has been demonstrated to display both chemopreventive and anticancer properties through inhibition of multiple signaling pathways^18^,^19^. Preclinical as well as clinical research shows that UA has tremendous potential to be developed into a potent anticancer drug^20^. The anti-leukemic properties of UA have been demonstrated *in vitro* and *in vivo*^21^,^22^. A previous study showed that p210 Bcr–Abl positive K562 cells, which are generally less sensitive to anticancer drugs, exhibited higher sensitivity to UA than non-CML cell lines such as HL-60 and U937^23^. Our previous study indicated that UA treatment induced apoptosis and increased JNK activation in K562 cells^24^. A recent publication described that UA treatment caused inactivation of the PI3K/Akt pathway by increasing PTEN gene expression in K562 cells^25^. However, the precise molecular mechanisms underlying UA sensitivity to growth inhibition of K562 cells as well as apoptosis induction remain in a large part unclear. In the present study, we demonstrate that UA induces apoptosis in K562 cells via Stat5 signaling, overcomes Imatinib resistance, and enhances both K562 and Imatinib-resistant K562/G01 cytotoxicity of Imatinib.

## Results

### Changes in expression of Bcl-2 family members in UA-induced apoptosis

**Figure 1:**
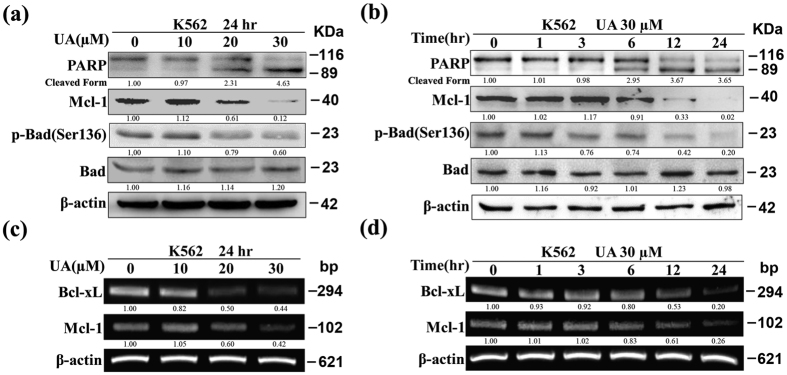
UA modulates expression of Bcl-2 family members in K562. Western blot analysis was conducted to determine protein expression of PARP, Mcl-1, p-Bad at Ser^136^, and Bad (**a**) after cells exposed to UA at various concentrations for 24 hr, and **(b)** after cells exposed to 30 μM UA for various times. RT-PCR was performed to determine mRNA expression of Bcl-xL and Mcl-1 (**c)** after cells exposed to UA at various concentrations for 24 hr, and (**d**) after cells exposed to 30 μM UA for various times. These experiments were repeated with similar results.

Our previous study showed that Bcl-xL protein expression was strikingly reduced in K562 cells exposed to UA^24^. In the present study, we found that after exposure of K562 cells to increasing concentrations of UA for 24 hr, protein levels of Mcl-1 and p-Bad at Ser136 were progressively declined (Fig. 1a). Significant alterations were detected at a UA concentration of 20 μM. Further analysis revealed that treatment with 30 μM UA for varying intervals resulted in a time-dependent down-regulation of Mcl-1 and p-Bad at Ser^136^, notable change being at 6 hr (Fig. 1b). The changes of Mcl-1 and p-Bad were roughly in parallel with PARP cleavage. No change in the total Bad level was observed. In consistence with protein expression, the mRNA levels of both Bcl-xL and Mcl-1 were significantly decreased in a dose- and time-dependent manner when cells underwent UA-induced apoptosis (Fig. 1c,d).

### UA-induced apoptosis involves decrease of Stat5a/b expression

**Figure 2:**
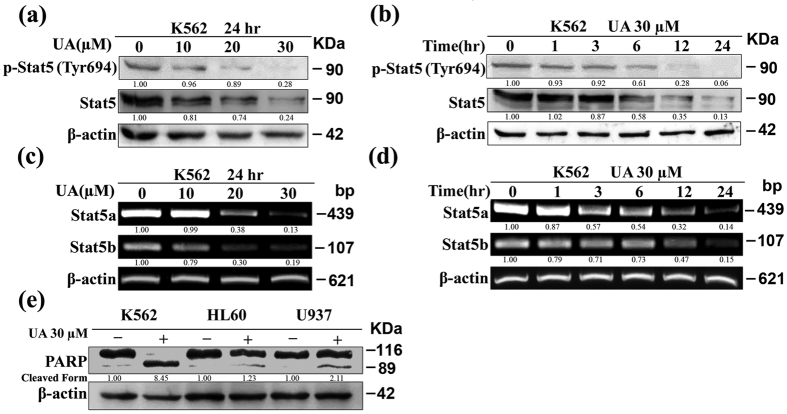
Stat5 inhibition by UA contributes to the sensitivity in K562. Western blot analysis was conducted to determine protein expression of p-Stat5 at Tyr^694^ and Stat5 (**a**) after cells exposed to UA at various concentrations for 24 hr, and (**b**) after cells exposed to 30 μM UA for various times. RT-PCR was performed to determine mRNA expression of Stat5a/b (**c**) after cells exposed to UA at various concentrations for 24 hr, and (**d)** after cells exposed to 30 μM UA for various times. (**e**) Western blot analysis was conducted to determine PARP cleavage in K562, HL-60, and U937 cells individually exposed to 30 μM UA for 24 hr. These experiments were repeated with similar results.

Considering that Bcl-xL is a target gene of Stat5^26^, we tested the ability of UA to modulate constitutive Stat5 activation. Figure 2a shows that the levels of p-Stat5 at Tyr^694^ progressively decreased after K562 cells were incubated with increasing concentrations of UA for 24 hr. Unexpectedly, the decrease in p-Stat5 was accompanied by an evident reduction of the amount of total Stat5 protein. Likewise, after exposure of K562 cells to 30 μM UA, the level of p-Stat5, along with total Stat5 protein, was reduced in a time-dependent manner (Fig. 2b). Results of RT-PCR in Fig. 2c,d are consistent with respective protein expression data: a dose- and time-dependent inhibition of Stat5a/b mRNA expression by UA occurred.

A previous study in leukemia cell lines demonstrated that K562 was more sensitive to UA-induced growth inhibition than HL-60 and U937 cell lines^23^. Consistently, when exposed to 30 μM UA under identical conditions for 24 hr, PARP was found to be largely cleaved in K562 cells, while only partial cleavage was observed in either HL-60 or U937 cells (Fig. 2e). Meanwhile, both HL-60 and U937 were found to express low Stat5 proteins and lack detectable levels of p-Stat5 in comparison with K562 cells (data not shown), in consistence with previously published reports^27^,^28^.

### UA-mediated Stat5 down-regulation and apoptosis involves induction of Gfi-1

**Figure 3:**
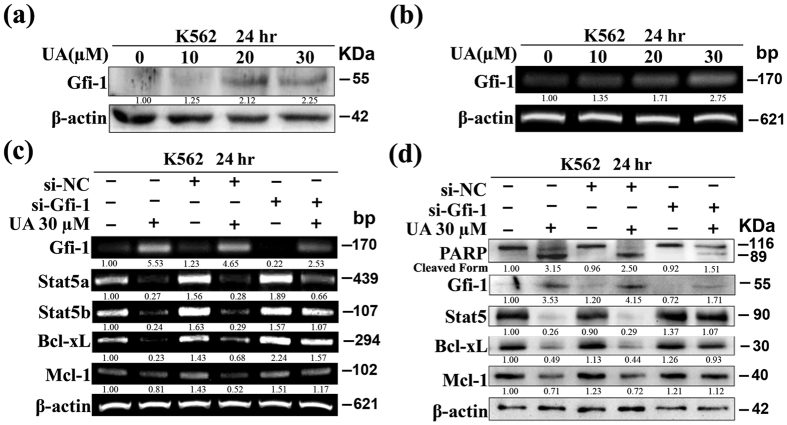
UA-induced apoptosis in K562 involves Gfi-1/Stat5b pathway. After cells exposed to UA at various concentrations for 24 hr, (**a**) Western blot analysis was conducted to determine protein expression of Gfi-1, and (**b**) RT-PCR was performed to determine mRNA expression of Gfi-1. After cells transfected with si-Gfi-1/si-NC and treatment with or without 30 μM UA for 24 h, (**c**) RT-PCR was performed to determine mRNA expression of Gfi-1, Stat5a/b, Mcl-1, and Bcl-xL, and **(d)** Western blot analysis was conducted to determine protein expression of PARP, Gfi-1, Stat5, Mcl-1, and Bcl-xL. These experiments were repeated with similar results.

The underlying regulatory mechanism for high expression of Stat5 in CML is still not fully understood. A recent study demonstrated that Stat5 is a directly repressed target of Gfi-1 in K562 cells^29^. To study the mechanism underlying UA-mediated decrease of Stat5 expression in K562 cells, we tested if UA was able to stimulate Gfi-1 expression in K562 cells. As expected, UA treatment led to a dose-dependent increase of both protein and mRNA expression of Gfi-1 in K562 cells (Fig. 3a,b).

To determine whether increased expression of Gfi-1 by UA was associated with the observed mRNA reduction in Stat5a/b, K562 cells were transfected with Gfi-1-siRNA to knockdown Gfi-1 expression, and then treated with or without 30 μM UA for 24 hr. Figure 3c shows that Gfi-1-siRNA completely abolished Gfi-1 expression in K562 with low Gfi-1 expression and evidently abrogated UA-induced Gfi-1 elevation. As expected, si-Gfi-1 recovered the mRNA expression of Stat5b, Bcl-xL, and Mcl-1 but had less impact on Stat5a in cells exposed to UA. On the other hand, in cells transfected with si-NC, UA treatment caused the same expression change patterns as in non-transfected cells. The RT-PCR results were confirmed by protein expression data. Moreover, the si-Gfi-1 evidently decreased the cleaved form of PARP induced by UA, whereas treatment by si-NC had little effect (Fig. 3d). FACS analysis also revealed that the si-Gfi-1 reduced the relative increase in apoptotic cells (data not shown). Taken together, the results suggested that the suppression by si-Gfi-1 could attenuate the pro-apoptotic effect of UA.

### UA-induced Stat5a/b down-regulation correlates with the decrease of Akt1/2 expression

**Figure 4:**
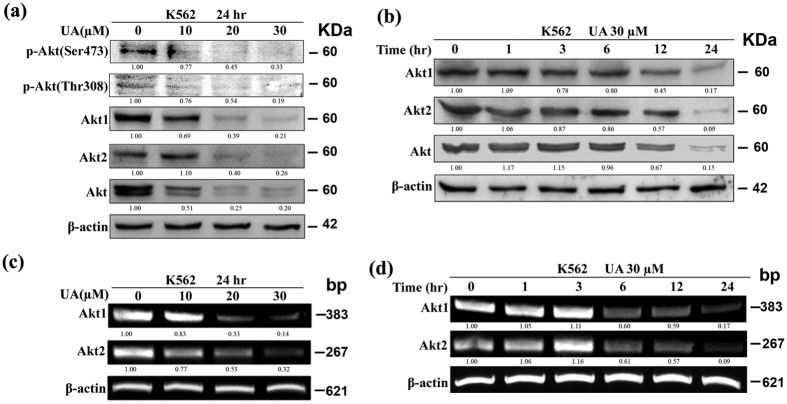
UA down-regulates expression of Akt1/2 in K562. Western blot analysis was conducted to determine protein expression of (**a**) p-Akt at Ser^473^, p- Akt at Thr^308^, Akt1, Akt2, and Akt after cells exposed to UA at various concentrations for 24 hr, and (**b**) Akt1, Akt2, and Akt after cells exposed to 30 μM UA for various times. RT-PCR was performed to determine mRNA expression of Akt1/2 **(c**) after cells exposed to UA at various concentrations for 24 hr, and (**d**) after cells exposed to 30 μM UA for various times. These experiments were repeated with similar results.

Considering that Akt signaling negatively regulates apoptosis through phosphorylation of Bad at Ser^136^ 30,31, we examined the effect of UA on activity of Akt in K562 cells. Consistent with a previous study^25^, we found that UA treatment inhibited p-Akt both at Ser^473^ and Thy^308^. Unexpectedly, we noticed the total Akt protein was reduced in UA-treated cells in a dose-dependent manner (Fig. 4a). Previously, Akt has been demonstrated to be a substrate of Caspase3. However, the pan-caspase inhibitor, Z-VAD-fmk, was not found to block the decrease of total Akt protein by UA (Data not shown). We next analyzed the amount of Akt1/2 proteins respectively by using antibodies that recognize each protein. As presented in Fig. 4a, UA treatment was found to inhibit expression level of each protein in a dose-dependent manner. Moreover, treatment with 30 μM UA resulted in a reduction of the expression of total Akt, Akt1, and Akt2 proteins in a time-dependent manner (Fig. 4b). RT-PCR results in Fig. 4c,d are consistent with protein expression data: UA was found to reduce Akt1/2 mRNA expression in a dose- and time-dependent manner.

**Figure 5:**
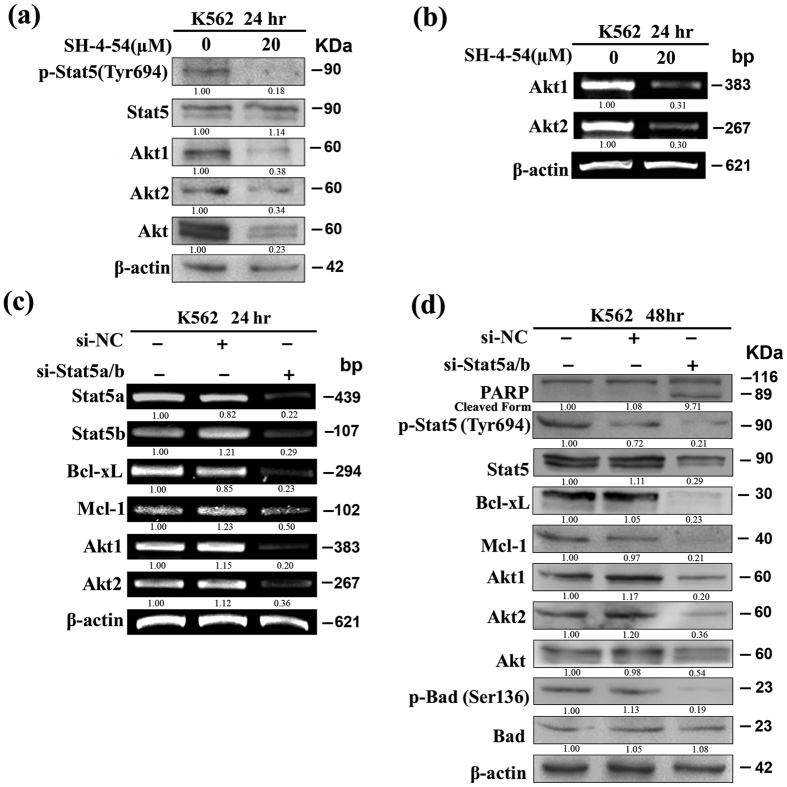
Akt1/2 expression is associated with Stat5 signaling in K562. After cells exposed to 20 μM SH-4-54 for 24 hr, (**a**) Western blot analysis was conducted to determine protein expression of p-Stat5 at Tyr^694^, Stat5, Akt1, Akt2, and Akt, and **(b)** RT-PCR was performed to determine mRNA expression of Akt1/2. After cells transfected with si-Stat5/si-NC, **(c)** RT-PCR was performed to determine mRNA expression of Stat5a/b, Bcl-xL, Mcl-1, and Akt1/2, and **(d)** Western blot analysis was conducted to determine protein expression of PARP, p-Stat5 at Tyr^694^, Stat5, Bcl-xL, Mcl-1, Akt1, Akt2, Akt, p-Bad at Ser^136^, and Bad. These experiments were repeated with similar results.

Akt1 gene was recently reported to be Stat5 transcriptional target in mammary epithelial cells^32^. In the light of our findings that UA-induced apoptosis was accompanied by a simultaneous decrease in Stat5 activity and Akt1/2 gene expression, we investigated whether Stat5 contributes to regulate Akt1/2 gene expression in K562 cells using Stat5 inhibitor SH-4-54. As presented in Fig. 5a, exposure to 20 μM SH-4-54 heavily curtailed the p-Stat5 at Tyr^694^ and had little effect on Stat5 protein level. Expectedly, SH-4-54 treatment causes evident down-regulation of Akt1/2 and Akt protein expression respectively (Fig. 5a). Consistently, RT-PCR revealed a marked decrease in Akt1/2 mRNA expression (Fig. 5b).

Next, the results obtained with SH-4-54 were validated upon silencing of Stat5 by means of RNA interference. As UA was found to down-regulate Stat5a/b mRNA, siRNA targeting Stat5a/b (si-Stat5) was used firstly. A significant down-regulation of Stat5a/b mRNA and total Stat5 protein could be achieved with si-Stat5 but no effect was produced by scrambled oligos (Si-NC) (Fig. 5c,d). A decrease in p-Stat5 at Tyr^694^ was also observed in interfered cells with the si-Stat5 (Fig. 5d). Consistent with previous reports^26^,^33^, Bcl-xL expression was inhibited dramatically and PARP cleavage was detected upon the silencing of Stat5. It is noteworthy that the down-regulation of Mcl-1 expression by si-Stat5 was not evident in comparison with Bcl-xL (Fig. 5c,d). Expectedly, cells interfered by si-Stat5 showed a significant decrease in expression of Akt1/2 mRNA (Fig. 5c). Moreover, administration of si-Stat5 resulted in a decrease in the levels of Akt1/2 protein, total Akt protein, and p-Bad at Ser^136^ while no change was observed in the levels of total Bad (Fig. 5d). In addition, MTT assay showed that cell viability in K562 cells was heavily suppressed by si-Stat5 but was almost not affected by Si-NC (data not shown).

To provide further information about the correlation between Stat5a/b deletion and Akt1/2 expression, siRNAs were generated for differentially knockdown human Stat5a and Stat5b. Similar to a previous study^12^, Stat5a siRNA (si-Stat5a) could be produced to inhibit only Stat5a with no effect on Stat5b, while attempts to obtain Stat5b siRNA of correspondingly similar effects was unsuccessful (data not shown). The Stat5a siRNA can inhibit Akt1 but not Akt2 mRNA expression in K562 cells (data not shown).

### UA induces apoptosis in K562/G01

**Figure 6:**
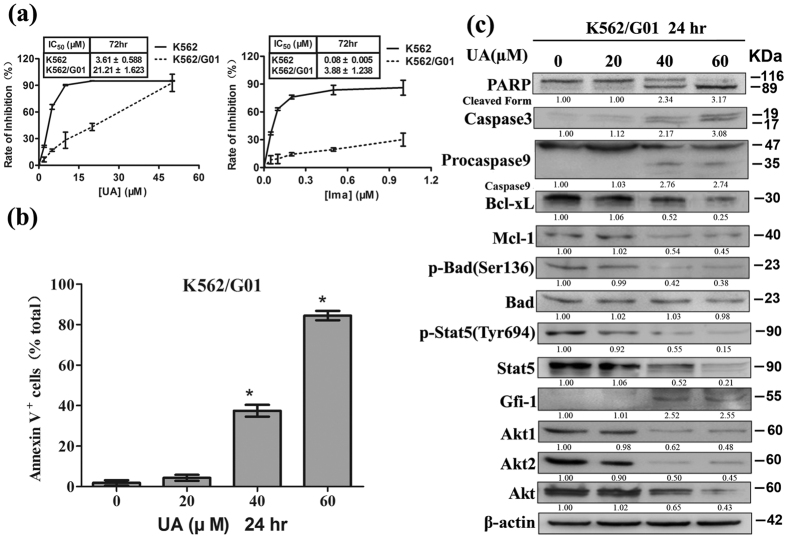
UA induces apoptosis in K562/G01. (**a**) After cells exposed to UA and Imatinib individually at various concentrations for 72 hr, cell viability was assessed by MTT (Error bars represent SD). The concentration required to cause a 50% reduction in cell viability (IC_50_) was calculated and shown as mean ± SD of mean in the inset box. After cells exposed to UA at various concentrations for 24 hr, **(b)** Annexin V-FITC/propidium iodide FACS was conducted to determine the percentage of apoptotic cells. Columns, mean; bars, SD; *P < 0.001 versus control, calculated by ANOVA, and **(c)** Western blot analysis was conducted to determine protein expression of PARP, Caspase3, Procaspase9, Bcl-xL, Mcl-1, p-Bad at Ser^136^, Bad, p-Stat5 at Tyr^694^, Stat5, Gfi-1, Akt1, Akt2, and Akt. These experiments were repeated with similar results.

Involvement of Stat5 in Imatinib resistance of CML cells prompted us to examine whether UA suppresses proliferation of Imatinib-resistant K562/G01. K562/G01 and K562 cells were individually exposed to UA or Imatinib at various concentrations for 72 hr. Cell viability was detected by MTT assay. The concentrations required to reduce cell viability by 50% (IC_50_) were calculated. As shown in Fig. 6a, treatment with either UA or Imatinib resulted in statistically significant differences in IC_50_ values between K562 and K562/G01. However, UA treatment resulted in decreases in cell viability in K562/G01 with an IC_50_ value of around 21.21 μM, which was around 6-fold more than that observed with parental K562. In contrast, K562/G01 was around 50- fold more resistant to the reference drug Imatinib than K562. Subsequent studies were performed to characterize the mechanisms underlying UA killed K562/G01. As shown in Fig. 6b, treatment with UA for 24 hr at doses beyond 40 μM caused statistically significant increases in the percentage of Annexin V^+^. Western blot analysis revealed that UA at the same doses resulted in strong increases in Caspase3 and cleavages of PARP and Procapase9, which suggested that UA induced cell death mainly by apoptosis. Moreover, the decrease in the expression of p-Stat5 at Tyr^694^, Stat5, Akt1, Akt2, and Akt individually in a dose-dependent manner was observed. Gfi-1 expression was also found to be elevated. Of Bcl-2 family proteins, Bcl-xL, Mcl-1 and p-Bad at Ser^136^ were down-regulated but Bad was unaffected (Fig. 6c).

### Synergistic activity of UA with Imatinib in K562 and K562/G01

**Figure 7:**
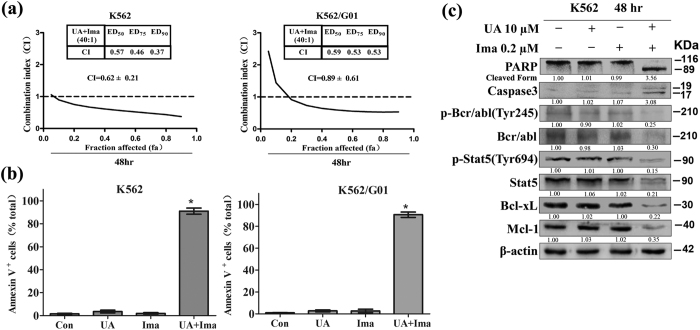
UA and Imatinib act synergistically in K562 and K562/G01. **(a)** MTT assay was performed to measure cell viability after cells exposed to various concentrations of UA and Imatinib alone or in combination at a fixed ratio for 48 hr. Chou-Talalay method was used to calculate CI values. Cell viability was expressed as fraction affected (fa). CI-values for ED_50_, ED_75_ and ED_90_ for the combination are shown, and the overall CI is given as mean ± SD of mean. After cells exposed to UA (K562:10 μM; K562/G01:20 μM) plus Imatinib (K562:0.2 μM; K562/G01:0.5 μM) for 48 hr, **(b**) Annexin V-FITC/propidium iodide FACS was performed to determine the percentage of apoptotic cells. Columns, mean; bars, SD; *P < 0.001 compared with either compound alone, calculated by ANOVA, and **(c)** Western blot analysis was conducted to determine protein expression of PARP, Caspase3, Bcl-xL, p-Bcr-abl at Tyr^245^, Bcr/Abl, p-Stat5 at Tyr^694^, Stat5, Bcl-xL, and Mcl-1 in K562 cells. These experiments were repeated with similar results.

Considering that Stat-5 signaling plays a key role in Bcr–Abl-mediated apoptosis resistance, we speculated that Stat-5 modulation induced by UA could increase the cytotoxic and apoptotic effects of Imatinib. To determine possible synergy between UA and Imatinib on cell proliferation, K562 and K562/G01 were individually treated with increasing doses of UA (K562: 0–40 μM; K562/G01: 0–80 μM) or Imatinib K562: 0–1 μM; K562/G01: 0–2 μM) alone or in combination at a fixed ratio (40:1) for 48 hr. MTT assay was performed to determine cell viability. Median Dose Effect analysis was used to calculate CI values. As can be seen in Fig. 7a, UA combined with Imatinib led to strong synergy along the entire dose-response curve, especially at moderate and higher concentrations of the two agents in both K562 and K562/G01. Moreover, a statistically significant increase in percentage of Annexin V^+^ populations was present in K562 cells treated with the combination of 10 μM UA and 0.2 μM Imatinib for 48 hr. Compared with K562 cells, K562G/01 displayed a statistically significant increase in percentage of Annexin V^+^ populations after exposure to 20 μM UA and 0.5 μM Imatinib (Fig. 7b). UA combined with Imatinib was found to induce apoptosis mainly as western blot analysis showing that evident cleavage fragments of PARP and Procaspase-3 appeared in K562 cells exposed to the combination for 48 hr (Fig. 7c).

Western blot analysis was also used to explore the molecular basis of apoptosis induction of UA in combination with Imatinib in K562 cells. As can be seen in Fig. 7c, co-exposure resulted in a decline in expression of p-Bcr/Abl at Tyr^245^, p-Stat5 Tyr^694^, Bcl-xL, and Mcl-1. It is noteworthy that combined treatment also resulted in a pronounced reduction of total protein of p210 Bcr/Abl and Stat5, whereas no evident alterations were observed in cells incubated with UA and Imatinib alone.

## Discussion

**Figure 8:**
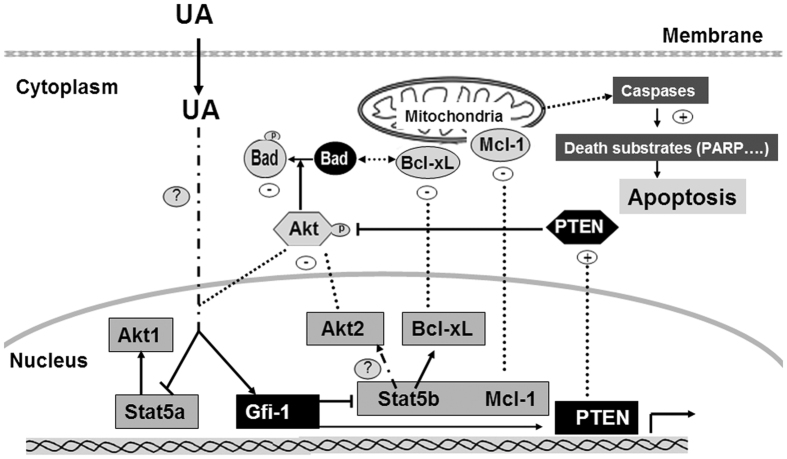
Schematic representation of UA effects on K562 cells. As described in the text, UA stimulates the expression of Gfi-1 and decreases Stat5a/b and Akt1/2 expression at transcriptional level. Of these effects SiRNA transfection studies reveal the regulation of Bcl-xL/Mcl-1/Bad for mitochondrial apoptosis by Gfi-1/Stat5/Akt pathway to be most important action.

In the present study, we provide evidence for a molecular mechanism whereby UA exerts at least part of its anticancer effects in CML cells (Fig. 8). We found that UA stimulated the expression of Gfi-1 and decreased Stat5a/b and Akt1/2 expression at transcriptional level in K562 cells. The knockdown of Gfi-1 by siRNA suppressed the induction of Gfi-1 by UA and reversed the inhibition of Stat5b expression. Additionally, UA-induced Stat5 inhibition correlated with suppression of Akt1/2 expression. The effect of Gfi-1/Stat5/Akt pathway by UA was associated with changes in expression of Bcl-2 family members. The results indicate the critical role of Gfi-1/Stat5/Akt pathway in the action of UA against K562 cells.

Multiple signaling pathways have been demonstrated to be involved in anti-cancer effect of UA^18^. Our results presented here are particularly novel because the data revealed, for the first time, that UA suppressed Stat5 activity via reducing Stat5a/b mRNA in Bcr-Abl-positive K562 cells. Previously, we have shown that UA increased JNK activity in K562 cells^24^. Whether increase of JNK activation by UA is linked to inhibition of Stat5 activation is not clear. Consistent with a previous report that K562 was more sensitive to UA than both HL-60 and U937^23^, we demonstrated increased cleavage of PARP in K562 than in HL-60 and U937 cells when they are treated by UA under the same condition (Fig. 2e). Given that both HL-60 and U937 cells lack Stat5 activity^27^,^28^, it is possible that the sensitivity of K562 to UA could be attributed to Stat5 inhibition. A previous study indicated that UA suppressed Stat3 activation but had no effect on Stat5 activity in multiple myeloma cells^34^. In the present study, we did not focus on the Stat3 activation by UA as it has been reported that activation of Stat3 could not be detected in K562 cells^14^. Collectively, the results indicate that the signaling pathways involved in UA action could be cell-type dependent.

Gfi-1 expression has been shown to play an important role for controlling the proliferation and survival of Bcr/Abl-expressing cells^35^. We demonstrated that UA clearly stimulated Gfi-1 expression in K562 cells and Gfi-1 siRNA attenuated UA-mediated apoptosis. We also provided evidence that the UA–induced inhibition of Stat5 expression involves induction of Gfi-1. Consistent with a recent report that Stat5b was Gfi-1-regulated gene in K562^29^, we observed that transfection with si-Gfi-1 reversed the Stat5b inhibitory effect of UA but had minimal action on Stat5a. In addition, the effect of UA on expression of apoptotic Bcl-xL and Mcl-1 was almost abrogated in si-Gfi-1-transfected cells. Consistent with a recent report that Mcl-1 expression could be a direct regulated target of Gfi-1^29^, we found that si-Stat5 transfection had minimal impact on Mcl-1 expression (Fig. 5d). On the basis of the previous reports that PTEN was induced in K562 cells in exposure to UA^25^ but also by ectopic expression of the Gfi-1^29^, we demonstrated that transfection with Gfi-1 siRNA also blocked the induction of PTEN by UA (data not shown). These results suggest that the inhibition of cell proliferation and induction of apoptosis in K562 cells are linked to Gfi-1 expression by UA. However, further work is still needed to determine regulatory mechanisms of Gfi-1 and Stat5a expression by UA in K562 cells.

Akt, a serine/threonine protein kinase, is present in three different isoforms named Akt1, Akt2, and Akt3 in mammals. Akt1 and Akt2 are expressed ubiquitously in nearly all tissue while Akt3 is expressed exclusively in the testis and neuronal tissue and up-regulated in some transformed cells^36^. Previous studies have demonstrated that Akt activity involved in UA-induced apoptosis in several types of cancer including leukemia^18^. In line with previous studies showing that pro-apoptotic factor Bad is rendered inactive due to its phosphorylation by Akt^30^,^31^, we found UA treatment led to down-regulation of p-Bad at Ser^136^, which suggests that inhibition of Akt/Bad pathway is also involved in UA-induced apoptosis in K562 cells. Interestingly, we found that UA clearly down-regulated both mRNA and protein levels of Akt1/2. To our knowledge, no study has yet described the effects of Akt expression by UA.

Despite a body of knowledge about the Stat5 signaling pathway being a major downstream of p210 Bcr-Abl in CML, there is less information available regarding Stat5 target genes regulating the proliferation and survival of CML cells. Our studies established a link between Stat5 signaling and expression of Akt1/2 in K562 cells. Upon inhibition of Stat5 expression by siRNA, Akt1/2 protein and p-Bad at Ser^136^ were significantly inhibited. Similar results were observed after cells exposed to Stat5 inhibitor. The results suggest that Akt activity in CML cells was regulated by Stat5 pathway. The PI3K/Akt pathway has been shown to participate in the Bcr-Abl-mediated resistance to apoptosis^37^. Our findings indicate that this event is partially associated with Stat5-dependent signaling. In addition, our findings mentioned above also support that the decrease of Akt activity in K562 cells by UA was directly impacted by increased PTEN. Therefore, it seems that UA –mediated Akt inhibitory activation involved in regulation of both gene expression and kinase activity.

Accumulated evidence indicates that Stat5a and Stat5b possess redundant and unique biological functions^38^,^39^,^40^,^41^. A new study suggests that Stat5a and Stat5b display a large degree of redundancy in regulating genes associated with cell proliferation and apoptosis^42^. In CML cells, antiapoptotic Bcl-xL and cell cycle regulator CyclinD1 have been identified to be Stat5-regulated genes^26^. Stat5b rather than Stat5a is recently thought to be responsible for Bcl-xL expression^33^. Consistent with a recent report that Akt1 is a transcriptional target of Stat5a in the lactating mammary epithelium, we found that si-Stat5a inhibited Akt1 but not Akt2 expression in K562 cells (data not shown). Further experiments will be necessary to determine whether Akt1/2 is a direct Stat5a/b target in CML cells.

Similar to the previous findings that Stat5 inhibitor can suppress growth and induce apoptosis in imatinib-resistant cells and enhance antitumor effect of TKI^16^,^17^, we demonstrated that UA exerted antileukemia action against imatinib-resistant K562 G/01 and UA combined with imatinib had synergistic effects on both K562 and K562 G/01. Alterations in protein expression caused by UA in K562 G/01 were similar to those observed in K562. However, a slightly higher dose of UA was found to be required in K562G/01 than in parental cells, suggesting that the possibility of non-specific action of other targets by UA cannot be excluded. Interestingly, combined treatment was found to result in a pronounced reduction of total protein of p210 Bcr/Abl in K562 cells. How this event is involved in UA action remains to be determined.

In conclusion, our study provided a novel mechanism for the antileukemia effect of UA on K562 cells. Future studies using other CML cell lines and animal models *in vivo* could ascertain whether the impact of UA on Gfi-1/Stat5/Akt pathway is a general proapoptotic-action mechanism and possibly shows novel therapeutic applications.

## Materials and Methods

### Reagents

UA (C_30_H_48_O_3_, MW = 456.68) was obtained from Sigma Chemical Co., St. Louis, USA. SH-4-54 was from Selleck Chemicals, Houston, USA. Imatinib (Imatinib Mesylate, Gleevec, STI571) was from Novartis Pharmaceuticals Basel, Switzerland. All reagents were prepared and used as recommended by their suppliers. Antibodies against PARP, Bcl-xL, Mcl-1, Bad, p-Stat5 at Tyr^694^, p-Akt at Ser^473^, p-Akt at Thy^308^, Akt, Akt1, Akt2, Procaspase9, Caspase3, c-Abl, and p-c-Abl at Tyr^245^ were from Cell Signaling Technology, Beverly, USA. The Antibody against Stat5 was from Zymed Laboratories Inc., South San Francisco, USA. Antibodies against p-Bad at Ser^136^, Gfi-1, and β-actin and HRP-conjugated secondary antibodies were from Santa Cruz Biotechnology Inc., Santa Cruz, USA.

### Cell lines and cell culture

K562, HL-60, and U937 cells were cultured in 10% fetal bovine serum/RPMI1640 medium (Gibco Invitrogen, Carlsbad, USA). K562/G01 cells obtained from the Institute of Hematology of the Chinese Academy of Medical Sciences (Tianjin, China) were maintained in media containing 4 μM Imatinib^43^. Prior to use in experiments, K562/G01 cells were cultured in imatinib-free medium for two weeks. All experiments were performed utilizing logarithmically growing cells (1 × 10^5^ cells/ml).

### Western blot analysis

A modified method as previously described was used^44^,^45^. Briefly, collected cells were lysed immediately in buffer supplemented with 1 mM Na_3_VO_4_ (Sigma Chemical Co., St. Louis, USA) and a protease inhibitor cocktail (Roche Molecular Biochemicals, Mannheim, Germany). Protein concentration was determined using Bradford Protein Quantitation Kit (Beyotime Biotechnology, Haimen, China). Equal amounts of protein were separated on SDS-PAGE and electroblotted to nitrocellulose membrane. After blocking, the blots were incubated with an appropriate dilution of specific antibodies and then were incubated with HRP-conjugated secondary antibodies. Blots were visualized using a chemiluminescence assay. β-actin was used as a loading control.

### RT-PCR

After cells were harvested, total RNA was extracted using Trizol reagent (Invitrogen, Life Technologies, Carlsbad, USA) according to the manufacturer’s instructions. Total RNA (2 μg) was reverse transcribed (RT) with MMLV reverse transcriptase (Invitrogen Life Technologies, Carlsbad, USA). PCR was performed using 2 × Taq PCR MasterMix (Tiangen Biotech, Beijing, China). The specific prime sequences listed in Supplementary information (Table S1) and the PCR reaction conditions were used as described previously^6^,^46^,^47^,^48^,^49^,^50^,^51^. The PCR products were electrophoresed on 1.5% agarose gels containing EB along with DNA markers. β-actin was used as an internal control.

### siRNA transfection

siRNA against Stat5 (si-Stat5) or Gfi-1 (si-Gfi-1) was used to inhibit endogenous Stat5 or Gfi-1 expression. A scrambled siRNA (si-NC) was used as a negative control. The siRNA sequences listed in Supplementary information (Table S2) were designed and synthesized by GenePharma Co, Ltd., Suzhou, China. A modified method for siRNA transfection as previously described was used^52^. Briefly, total of 3 × 10^6^ K562 cells were suspended in 600 μl GenePulser Electroporation Buffer (Bio-Rad Laboratories, Hercules, USA) with each siRNA at the final concentration of 133 nm. Cells were electroporated using a Gene Pulser Xcell (Bio-Rad Laboratories, Hercules, USA) at 152 V and 1000 μF. Six hours after pulses, fresh RPMI1640 containing 10% FBS was added. At 24 hr after the initiation of transfection, cells were harvested for RT-PCR or UA treatment. Western blot was performed after the transfected cells were cultured for 48 hr.

### MTT assay

The viability of cells was estimated using MTT (Sigma Chemical Co., St. Louis, USA) assay described previously^53^. Briefly, 10 μl of MTT solution (5 mg/ml in ddH_2_O) was added to each well. The plates were then incubated for 4 hr at 37 °C. Intracellular formazan crystals were dissolved by addition of 100 μl of isopropanol-HCI-SDS solution to each well. After overnight incubation at 37 °C, the optical density of the samples was determined at 570 nm. Rate of inhibition was calculated by using the equation: Rate of inhibition = (Ac-At)/Ac × 100, where At and Ac represent the absorbance in treated and control cultures, respectively.

### Annexin V-FITC/propidium iodide FACS

Apoptotic rates were analyzed by flow cytometry using a commercially available Annexin V-FITC/propidium iodide (PI) apoptosis detection kit (KeyGen Biotech Co. Ltd., Nanjing, China). Staining was performed according to the manufacturer’s instruction, and flow cytometry was conducted on a BD Accuri C6 flow cytometer (BD Biosciences, San Jose, USA). In this assay, Annexin V^+^/PI^−^ populations are early apoptotic cells, Annexin V^+^/PI^+^ populations are late apoptotic (secondary necrotic form) and primary necrotic cells.

### Statistical and densitometric analysis

In MTT assay and FACS analysis, values represent the mean ± standard deviation (SD) around the mean from three separate studies performed in triplicates. The IC_50_ values were determined and compared by Student’s t-test using Graphpad software. FACS data were analyzed by ANOVA, using SPSS 9.0 software. P values < 0.05 were considered to be statistically significant. The interaction between UA and Imatinib was analyzed by calculating the combination index (CI) values according to the Chou-Talalay method^54^ using Software Spss 13.0. A CI less than 1 indicates synergy, a CI = 1 indicates an additive effect, and a CI more than 1 indicates antagonism between the two agents. Densitometric quantification of Western blots and PCR-amplified products was performed using Image J software. The data presented below the bands are fold changes over the control after being normalized to that of the corresponding β-actin bands.

## Additional Information

**How to cite this article**: Lin, Z. *et al.* Ursolic acid-mediated apoptosis of K562 cells involves Stat5/Akt pathway inhibition through the induction of Gfi-1. *Sci. Rep.* **6**, 33358; doi: 10.1038/srep33358 (2016).

## Supplementary Material

Supplementary Information
