# Supplementary material for: Ursolic acid-mediated apoptosis of K562 cells involves Stat5/Akt pathway inhibition through the induction of Gfi-1
Source: Sci Rep. 2016 Sep 16;6:33358. doi: 10.1038/srep33358 (PMC5025887; doi:10.1038/srep33358)
Supplement: Supplementary Information [file srep33358-s1.doc]

**Ursolic acid-mediated apoptosis of K562 cells involves**

**Stat5/Akt pathway inhibition through the induction of Gfi-1**

Ze Lin^1^, Jikai Jiang^1^, Xiao-Shan Liu^2^

^1^ Department of Biochemistry, Shantou University Medical College, Shantou, 510451, China

^2^ Department of Biochemistry, Guangzhou Medical University, Guangzhou, 511436, China

**SUPPLEMENTARY INFORMATION**

**SUPPLEMENTARY TABLES**

**Table S1 Primer sequences for RT-PCR**

| Gene | 5'-3' sequences Forward/Reverse |
| --- | --- |
| Bcl-xL | TTCAGTGACCTGACATCCCAG / TGCATTGTTCCCATAGAGTTCC |
| Mcl-1 | GGTTTTGGTGGTGGTGGTGGTTGG / GGTGGTGGTGGTTGGTTAAAAGTC |
| Stat5a | CCCGGAACGCAACCTGTGGAACC / GGGGCGAGAGGCGGGAGTCAAGA |
| Stat5b | GTAAACCATGGCTGTGTGGA / AAATAATGCCGCACCTCAAT |
| Gfi-1 | TCCAGCCTCGGAGAAGTC / TCGCAGAAGAGGCCCAGG |
| Akt1 | GCTGGACGATAGCTTGGA / GATGACAGATAGCTGGTG |
| Akt2 | GGCCCCTGATCAGACTCTA / TCCTCAGTCGTGGAGGAGT |
| β-actin | ACACTGTGCCCATCTACGAGG / AGGGGCCGGACTCGTCATACT |

**Table S2 Sequences of the siRNAs**

| Gene | 5'-3' sequences Forward/Reverse |
| --- | --- |
| Stat5a/b | CCAAGGAGAACCUCGUGUUTT / AACACGAGGUUCUCCUUGGTT |
| Gfi-1 | GCUCCUACAAGUGCAUCAATT / UUGAUGCACUUGUAGGAGCTT |
| Negative control | UUCUCCGAACGUGUCACGUTT / ACGUGACACGUUCGGAGAATT |
